# The combination of *Artemisia princeps Pamp*, *Leonurus japonicas Houtt*, and *Gardenia jasminoides Ellis* fruit attenuates the exacerbation of energy, lipid, and glucose by increasing hepatic PGC-1α expression in estrogen-deficient rats

**DOI:** 10.1186/s12906-016-1109-x

**Published:** 2016-05-23

**Authors:** Hye Jeong Yang, Min Jung Kim, Dae Young Kwon, Bo Reum Moon, A. Reum Kim, Suna Kang, Sunmin Park

**Affiliations:** Food Functional Research Division, Korean Food Research Institutes, Sungnam, Korea; Department of Food and Nutrition, Obesity/Diabetes Center, Hoseo University, Asan, Korea; Department of Food and Nutrition, Hoseo University, 165 Sechul-Ri, BaeBang-Yup Asan-Si, Asan, ChungNam-Do 336-795 South Korea

**Keywords:** Estrogen deficiency, Glucose, Insulin, Lipid profiles, PGC-1α

## Abstract

**Background:**

*Artemisia princeps Pamp* (APP), *Leonurus japonicas Houtt* (LJH), and *Gardenia jasminoides Ellis* fruit (GJE) have been traditionally used in East Asia to treat women’s diseases related to reproductive system. They may attenuate the deterioration of energy, lipid, glucose and bone metabolism by estrogen deficiency. The present study explored the combination of APP, LJH, and GJE to overcome the symptoms of estrogen deficiency and the mechanism was explored.

**Methods:**

Ovariectomized (OVX) rats were divided into five groups and fed high-fat diets supplemented with 2 % dextrin (control), 2 % APP, 2 % APP + LJH (15:5), APP + LJH + GJE (10:5:5) or 17β-estradiol (30 μg/kg bw/day) for 8 weeks. After 8 weeks of their consumption, energy, lipid, glucose and bone metabolisms were investigated and hepatic insulin signaling and fatty acid metabolism were determined.

**Results:**

APP + LJH + GJE, but not APP itself, improved energy metabolism and attenuated a decrease in energy expenditure by the same amount as estrogen. Moreover, APP + LJH + GJE reduced visceral fat and intramuscular fat and increased lean body mass measured by DEXA by as much as the positive-control. APP itself suppressed increased LDL cholesterol and triglyceride levels in OVX rats and APP + LJH + GJE alleviated dyslipidemia in OVX rats. Overnight-fasted serum insulin levels and HOMA-IR were reduced in the descending order of APP, APP + LJH, APP + LJH + GJE, positive-control in OVX rats. APP and APP + LJH elevated insulin secretion in the 1st part of OGTT to decrease serum glucose levels while APP + LJH + GJE reduced serum glucose levels without increasing serum insulin levels during OGTT. APP + LJH + GJE decreased insulin resistance during ITT in OVX rats more than the positive-control. The APP + LJH + GJE group exhibited increased hepatic peroxisomal proliferator-activated receptor-γ coactivator-1α expression, which increased the number of genes involved in fatty acid oxidation and decreased fatty acid synthesis. Hepatic insulin signaling (pAkt and pGSK-1β) was also potentiated to reduce phosphoenolpyruvate carboxykinase proteins.

**Conclusion:**

The combination of APP + LJH + GJE attenuated various menopausal symptoms in OVX rats. Thus, it may have potential as a therapeutic agent for the treatment of postmenopausal symptoms.

## Background

Menopause is a transitional phase from a reproductive to a non-reproductive phase in a woman’s life. Various symptoms are common during menopause and hot flashes, cognitive changes, anxiety and depression are included [1]. The symptoms are not deadly diseases but reduce the quality of a woman’s life. In addition to menopausal symptoms, estrogen deficiency results in the decrease of energy, glucose, lipid and bone metabolism by reducing peroxisome proliferator-activated receptor-γ coactivator (PGC)-1α expression in various tissues [2]. This impairment may not influence daily life as much as menopausal symptoms but can eventually develop into metabolic diseases such as obesity, dyslipidemia, type 2 diabetes, osteoarthritis, and osteoporosis [3]. Therefore, the deterioration of metabolism needs to be prevented and/or delayed in post-menopausal women.

Menopause occurs due to the marked decrease of female hormones, especially estrogen. Hormone replacement therapy is effective for menopausal symptoms but is a limited option due to increased health risks for breast cancer, cardiovascular diseases, and dementia [4]. There is growing interest in alternative treatments for menopausal symptoms. Plant extracts such as lineseeds, red clover, St. John’s wort, hop or black cohosh are most frequently used as phytochemical therapy [3, 5]. They contain some phytoestrogens, which have a similar chemical structure as estrogen and exhibit estrogenic or anti-estrogenic effects. There is growing evidence that herbs containing phytoestrogens have the potential to reduce menopausal symptoms without health risks, unlike hormone replacement therapy [5]. However, many herbal remedies do not have sufficient efficacy to reduce the symptoms of post-menopausal women [5]. It is necessary to explore alternatives to attenuating menopausal symptoms without adverse effects to improve women’s quality of life.

Various herbs were used traditionally to improve women’s health as menopausal symptoms were not recognized in the past. Relatively recent studies have shown that these herbs attenuate menopausal symptoms [6, 7] and some are commercially available. However, they only improve some post-menopausal symptoms, not all. Thus, better herbal treatments to alleviate menopausal symptoms need to be explored. Many herbs contain phytoestrogen, but they have different functionalities and a combination of herbs may have better efficacy in alleviating menopausal symptoms. Among them, *Artemisia princeps Pamp* (APP; Ganghwayakssuk or mugwort), *Leonurus japonicas Houtt* (LJH; Chinese motherworth), and *Gardenia jasminoides Ellis* (GJE; Cape Jasmine) are traditionally used to improve women’s health in East Asia including Korea. The major components of APP are eupatilin and jaceosidin, which are reported to reduce inflammation [8, 9]. LJH was mainly used for treating menoxenia, dysmenorrhea, amenorrhea, lochia, body edema, oliguresis, sores, ulcerations and other diseases in women in East Asia [10]. Pharmacological studies have demonstrated that the active components in LJH possess various functionalities such as cardioprotective, anti-oxidative, anti-cancer, analgesic, anti-inflammatory, neuroprotective and antibacterial actions [10]. Stachydrine is the main component of Chinese motherwort and is used as the official indicator to monitor its quality [11]. In addition, GJE has been reported to ameliorate hyperglycemia, hypertension, cerebral ischemia and dyslipidemia [12, 13]. It contains geniposide, ursolic acid, crocin and genipin. Geniposide and ursolic acid have the potential to inhibit glycogenolysis to increase glucose levels in the circulatory system, and improve lipid metabolism [14]. In addition, GJE is reported to protect liver function and neuronal cell death by activating anti-inflammatory activity through geniposide [15]. Although both mugwort and motherwort are known to be improve women’s reproductive system to reduce primary dysmenorrhea and GJE has been shown to improve glucose and lipid metabolism, they have not been studied for the purpose of alleviating post-menopausal symptoms.

We were interested in APP for alleviating menopausal symptoms but it might not be sufficient to attenuate the deterioration of energy, glucose, lipid, and bone metabolism in estrogen deficient conditions. LJH and GJE were therefore combined with APP to increase the efficacy for anti-menopausal symptoms. We hypothesized that the mixture of APP, LJH and GJE would ameliorate the reduction in energy, glucose, lipid and bone metabolism caused by estrogen deficiency. We tested the hypothesis using ovariectomized rats and explored their mechanisms.

## Methods

### Preparation of APP, LJH and GJE water extracts

APP, LJH and GJE were grown in Korea and APP leaves, LJH leaves and GJE fruit were purchased from Ganghwa Sajabal Ssook Inc. (Ganghwa, Korea) in 2013. They were identified by Dr. Byung Seob Ko (Korean Institutes of Oriental Medicine, Daejeon, Korea), and a voucher specimen (No. 2013–04, 2013–05 and 2013–06) deposited at the herbarium of Korean Institutes of Oriental Medicine. Dried and ground APP leaves, LJH leaves and GJE fruits (2 kg) were extracted three times by refluxing with water at 80 °C for 3 h, after which the filtered extracts were lyophilized. The yields of APP leaves, LJH leaves and GJE fruit were 14.8, 15.5 and 20.4 %, respectively. Each of the dried extract was dissolved in methanol. The total phenolic compound contents were then measured using Folin-Ciocalteu reagent [16] and expressed as mg gallic acid equivalents · g^−1^. The extracts were dissolved in ethanol and total flavonoid contents were measured using the modified methods described previously [17]. Rutin was used as a standard.

### Analysis of bioactive compounds

The analyses were performed using an Acquity UPLC system (Waters, Miliford, MA, USA) with an Acquity UPLC BEH C18 column (2.1 mm × 100 mm, 1.7 μm). Mass spectrometric analyses were operated using a Waters Xevo TQ triple-quadrupole mass spectrometer in electrospray ionization (ESI) mode. Individual APP, LJH and GJE extracts were dissolved in methanol for quantifying the indicator compound. For eupatilin and jaceosidin analysis in APP, pyridine was added to methanol containing APP (1:10, v/v) and the mixture was injected into the UPLC. An isocratic mobile phase of 70 % methanol and 0.1 % TFA was used with a flow rate of 1.5 mL/min. The column temperature was 35 °C, the injection volume was 20 μL, and UV detection was performed at 285 nm.

For geniposide and ursolic acid analysis in GJE, there was an isocratic mobile phase of acetonitrile: methanol: water (45:45:10 v/v/v) with a flow rate of 0.4 mL/min. The injection volume was 5 μL and column temperature was maintained at 35 °C. The tandem MS was operated in negative ESI mode, and processed using MassLynx 4.1 (Waters) software. Quantification was performed using a single ion monitoring (SIM) mode of *m/z* 445.4 for ursolic acid. The detector was operated with a cone voltage of 35 V and a capillary voltage of 3.0 kV. The source temperature was set at 150 °C, while the desolvation flow rate and gas temperature were set at 800 L/h and 500 °C, respectively.

For stachydrine analysis in LJH, the mobile phase was composed of (A) 0.1 % formic acid aqueous solution and (B) 0.1 % formic acid in acetonitrile, at a flow rate of 0.6 mL/min. The conditions were as follows: initial condition of 99 % A, 0- 3 min at 99–70 % B, 3–5 min at 99 % A. The injection volume was 2 μL, column temperature was kept at 30 °C and the total run time was 5 min. The mass spectrometer was operated in positive ESI mode and scanned using the multiple reaction monitoring (MRM) mode. The MRM transitions were monitored at m/z 144.1 → 58.1, 84.1 for stachydrine. The voltage of capillary, cone and collision energy was set at 3.5 kV, 33 V and 22 V, respectively. The gas flow for desolvation and cone was set at 800 and 50 L/h.

### Experimental animals and design

Ovariectomy (OVX) was performed on female Sprague–Dawley rats aged 8–10 weeks (219 ± 13 g) and they were housed individually in stainless steel cages in a controlled environment (23 °C with a 12-h light and dark cycle). All surgical and experimental procedures were approved by Hoseo University Animal Care and Use Review Committee (2013–04), which reviewed the procedures based on NIH Guidelines. The OVX rats were randomly separated into 5 groups. They freely consumed water and their assigned respective diets for 8 weeks. The high fat diet was a modified semi-purified AIN-93 formulation [18] consisting of 40 energy percent (En%) carbohydrates, 20 En% protein, and 40 En% fats. The major carbohydrate, protein and fat sources were starch plus sugar, casein (milk protein), and lard (CJ Co, Seoul).

Sixty OVX rats were randomly divided into five dietary groups: control, APP, APP + LJH, APP + LJH + GJE and 17β-estradiol (positive-control group). Their diets contained 2 % dextrose, 2 % APP, 2 % APP + LJH (15:5) or 2 % APP + LJH + GJE (10:5:5) in the high fat diet, respectively. The dosage of herb extracts used in the present study is equivalent to approximately 3–5 g/day for human usage. The diet for the positive-control group contained 30 μg/kg body weight of 17β-estradiol + 2 % dextrose.

### Tail skin temperature measurement

Tail skin temperature was measured using an infrared thermometer (BIO-152-IRB, Bioseb, Chaville, France) designed for small rodents at the 1th and 8th weeks of the experimental periods during the sleep cycle. Three measurements were made 10 min apart and the average value for the animal was used as a single data point for each week [19].

### Energy expenditure by indirect calorimetry

After 7 weeks of the assigned diet, the rats were fasted for 6 h before the beginning of the dark phase and energy expenditure was measured. Energy expenditure was assessed by indirect calorimetry measuring average oxygen consumption (VO_2_) and average carbon dioxide production (VCO_2_): a rat was placed in the metabolic chambers (airflow = 800 ml/min) with a computer-controlled O_2_ and CO_2_ measurement system (Biopac Systems Inc., Goleta, CA) for 30 min. The respiratory quotient (RQ) and resting energy expenditure were calculated using the equations described by Niwa et al. [19]. After the experiment, data were averaged over 1 min intervals and VO_2_ and VCO_2_ values were corrected for metabolic body size (kg^0.75^) [20]. The amounts of carbohydrate and fat oxidation were calculated from non-protein oxygen consumption as were their relative oxidative proportions and the amount of oxygen consumed per gram of substrate oxidized [20].

### Oral glucose tolerance test (OGTT) and insulin tolerance test (ITT)

Two days after measuring energy expenditure, an OGTT was conducted in overnight-fasted animals by orally administering 2 g glucose/kg body weight. After glucose loading, blood samples were taken by tail bleeding at 0, 10, 20, 30, 40, 50, 60, 70, 80, 90, and 120 min to measure serum glucose levels with a Glucose Analyzer II (Beckman, Palo Alto, CA) and serum insulin levels were measured at 0, 20, 40, 60, 90 and 120 min with a radioimmunoassay kit (Linco Research, Billerica, MA). The average of the total areas under the curves of the serum glucose and insulin levels during the OGTT was calculated by the trapezoidal rule. In addition, since insulin is released in two phases after glucose, serum glucose and insulin levels were divided into two parts [21, 22]. In glucose tolerant condition, insulin release is peak at 15–30 min after glucose road (early phase) but in glucose intolerant condition, insulin release is delayed. In the present study, the early phase was defined as 0–40 min and the 2nd phase was 40–120 min.

Three days after OGTT, an ITT was conducted after the withdrawal of food for 6 h. Serum glucose levels were measured every 15 min for 90 min after intraperitoneal insulin injection (0.75 U/kg body weight). Serum glucose levels were measured by collecting blood through tail bleeding. Afterwards, food and water was freely provided for two days and then they were overnight-fasted to be scarified.

### Body composition measurement

At the day scarifying the rats, body composition was measured by dual-energy X-ray absorptiometry (DEXA) using an absorptiometer (pDEXA Sabre; Norland Medical Systems Inc., Fort Atkinson, WI). Briefly, a densitometer was calibrated with a phantom supplied by the manufacturer on a daily basis [6]. The animals were laid in a prone position, with their hind legs maintained in external rotation with tape after the anesthetization with ketamine and xylazine (100 and 10 mg/kg body weight, respectively). Hip, knee and ankle articulations were in 90° flexion and body composition was measured. After the completion of scanning, bone mineral density (BMD) was determined in the right femur and lumbar spine. The pDEXA was equipped with the appropriate software for assessment of body composition in small animals. Similarly, abdominal fat mass and lean mass in abdomen, hip and leg were measured by DEXA.

After finishing DEXA analysis, blood samples were collected from the tail bleeding. After centrifugation of the blood, lipid profiles in circulation were determined by measuring serum levels of triglyceride, total cholesterol, and HDL cholesterol using the appropriate colorimetry kits (Asan Pharm., Seoul, Korea). In addition, liver function was measured by aspartate aminotransferase (AST) and alanine aminotransferase (ALT) in the circulation using colorimetry kits (Asan Pharm.). Serum leptin levels were determined using a radioimmunoassay kit (Linco Research). Insulin resistance was determined using the homeostasis model assessment estimate of insulin resistance (HOMA-IR) [HOMA-IR = fasting insulin (μIU/ml) × fasting glucose (mM) / 22.5].

After drawing blood, human regular insulin (unmodified insulin; 5 U/kg body weight) was injected through the inferior vena cava. Ten min later, the rats were killed by decapitation and tissues such as liver and gastrocnemius and quadriceps muscles were rapidly collected, frozen in liquid nitrogen, and stored at −70 °C for further experiments. Epididymal and retroperitoneal fat mass and uteruses were then excised and weighed. Uterus index was calculated as uterus weight divided by body weight.

### Triglyceride contents in the liver and skeletal muscles

Triacylglycerol was extracted from the livers and gastrocnemius and quadriceps muscles with chloroform-methanol (2:1, vol/vol) and resuspended in pure chloroform [23]. After evaporating the chloroform, the residues were suspended with PBS with 0.1 % triton X-100 and the suspension was sonicated and boiled for 5 min. The triacylglycerol contents of the suspensions were assayed using a Trinder kit (Asan).

### RNA isolation and reverse transcription polymerase chain reaction (RT-PCR)

The livers of four rats were randomly selected from each group. Total RNA was isolated from the liver using a monophasic solution of phenol and guanidine isothiocyanate (Trizol reagent, Gibco-BRL, Rockville, MD), followed by extraction and precipitation with isopropyl alcohol. The cDNA was synthesized from equal amounts of total RNA with superscript III reverse transcriptase, and the contents of cDNA was enlarged by PCR with high fidelity Taq DNA polymerase. Equal amounts of cDNA were mixed with sybergreen mix and analyzed using a realtime PCR machine (BioRad, Richmond, CA). The expression level of the gene of interest was corrected to that of the house keeping gene, β-actin. The primers used to detect rat peroxisome proliferator-activated receptor-gamma coactivator (PGC)-1α, carnitine palmitoyltransferase-1 (CPT-1), acetyl CoA carboxylase (ACC), sterol regulatory element-binding protein-1c (SREBP-1c), fatty acid synthase (FAS), and β-actin genes were described previously [7].

### Immunoblot analysis

The frozen livers of four rats were lysed with a 20 mM Tris buffer (pH 7.4) containing 2 mM EDTA, 137 mM NaCl, 1 % NP40, 10 % glycerol, 12 mM α-glycerol phosphate and protease inhibitors. Liver lysates containing equal amounts of protein (30–50 μg) were resolved by SDS-PAGE, and immunoblotting was performed with specific antibodies against phosphorylated Akt and glycogen synthase kinase-1β (GSK-1β) and Akt, GSK-1β, phosphoenolpyruvate carboxykinase (PEPCK) and β-actin. The intensity of protein expression was determined using Imagequant TL (Amersham Biosciences, Piscataway, NJ). Three sets of two samples per group were evaluated (*n* = 6).

### Statistical analysis

All results are expressed as means ± standard deviations. Statistical analysis was performed using the SAS software (SAS institutes, Cary, NC, USA). The variables that measured at different time points were analyzed with two-way repeated measures analysis of variance (ANOVA) with time and group as independent variables and interaction term between time and group. One-way ANOVA was used to determine the group (APP, APP + LJH, APP + LJH + GJE, positive-control and control groups) effect when the results were measured once at the end of experiment. Significant differences in the main effects among the groups were identified by Tukey’s test at *p* < 0.05.

## Results

### The contents of total phenolic compounds, flavonoids, and bioactive components

Total polyphenol and flavonoids contents were about 3 fold higher in GJE compared to APP and LJH (Table 1). The indicator compounds in each water extract were eupatilin and jaceosidin in APP, stachydrine in LJH and geniposide and ursolic acid in GJE. The amount of each of these compounds in the water extract was sufficient to use them as indicator compounds (Table 1).

**Table 1:** The contents of total polyphenols, total flavonoids and indicator compounds in *Artemisia princeps Pamp*, *Leonurus japonicas Houtt, Gardenia jasminoides Ellis* fruit water extracts

		Total polyphenols (mg/g)	Total flavonoids (mg/g)
*Artemisia princeps Pamp* (APP)		7.4 ± 0.6	3.3 ± 0.2
*Leonurus japonicas Houtt* (LJH)		6.8 ± 0.4	2.4 ± 0.1
*Gardenia jasminoides Ellis* fruit (GJE)		21.2 ± 0.7	18.4 ± 0.9
	Contents (mg/g)		Contents (mg/g)
Eupatilin in APP	1.34 ± 0.11	Geniposide in GJE	4.72 ± 0.33
Jaceosidin in APP	0.88 ± 0.09	Ursolic acid in GJE	0.02 ± 0.01
Stachydrine in LJH	1.50 ± 0.23		

Values are means ± SD (*n* = 3)

### Tail skin temperature

**Fig. 1:**
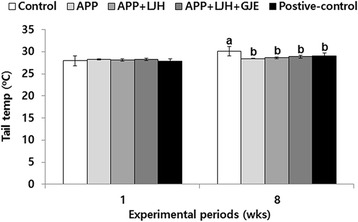
Tail skin temperature at 1st and 8th weeks of experimental period. Control, OVX rats fed a high-fat diet with 2 % dextrin; APP, OVX rats fed a high fat diet with 2 % *Artemisia princeps Pamp* water extract; LJH, OVX rats fed a high fat diet with 2 % *Leonurus japonicas Houtt* water extract; GJE, OVX rats fed a high fat diet with 2 % *Gardenia jasminoides* Ellis water extract; positive-control, OVX rats fed a high fat diet with 30 μg/kg body weight 17β-estradiol + 2 % dextrose. At 1st and 8th weeks, tail skin temperature was measured using infrared thermometer. Bars and error bars represent means ± SD (*n* = 12). ^a,b^ Significantly different among all groups at *p* < 0.05

Estrogen deficiency elevates skin temperature due to a vasomotor disorder and is known to increase tail skin temperature in OVX rats. None of the treatments changed the tail skin temperature in the first week, but rats in the control group exhibited higher tail skin temperature at week 8 than those in the positive-control group (Fig. 1). APP, APP + LJH and APP + LJH + GJE suppressed the increase in OVX rats as much as the positive-control (Fig. 1).

Estrogen deficiency is also reported to reduce the size of the uterus. The uterine index was lower by about 3 folds in the control rats in comparison to the positive-control rats (Table 2). It indicated that APP, APP + LJH and APP + LJH + GJE have no uterine proliferation unlike estrogen treated rats (positive-control).

**Table 2:** Metabolic parameters related to energy metabolism at the end of experimental periods

	Control (*n* = 12)	APP (*n* = 12)	APP + LJH (*n* = 12)	APP + LJH + GJE (*n* = 12)	Positive-control (*n* = 12)
Body weight (g)	355 ± 18^a^	360 ± 25^a^	362 ± 19^a^	340 ± 19^b^	333 ± 18^b^
Body weight gain (g)	96.9 ± 7.7^a^	99.1 ± 9.4^a^	101.2 ± 7.3^a^	70.3 ± 7.3^b^	74.1 ± 7.9^c^
Peri-uterine fat (g)	11.2 ± 1.1^a^	8.9 ± 0.9^b^	9.2 ± 0.9^b^	6.6 ± 0.7^c^	6.1 ± 0.7^c^
Ratio of peri-uterine fat and body weight	0.032 ± 0.007^a^	0.025 ± 0.004^b^	0.025 ± 0.005^b^	0.019 ± 0.004^c^	0.018 ± 0.004^c^
Retroperitoneum fat (g)	13.3 ± 1.4^a^	12.2 ± 1.3^a^	13.0 ± 1.4^a^	8.7 ± 1.0^b^	8.2 ± 0.8^b^
Ratio of retroperitoneum fat and body weight	0.037 ± 0.008^a^	0.034 ± 0.005^a^	0.036 ± 0.007^a^	0.026 ± 0.005^b^	0.025 ± 0.005^b^
Uterine index	0.56 ± 0.06^c^	0.56 ± 0.06^c^	0.62 ± 0.07^c^	0.91 ± 0.09^b^	1.53 ± 0.14^a^
Overnight fasted leptin levels (ng/mL)	3.4 ± 0.6^a^	3.8 ± 0.6^ab^	3.9 ± 0.6^ab^	4.1 ± 0.6^b^	4.3 ± 0.7^b^

Control, OVX rats fed a high-fat diet (OVX-CON) with 2 % dextrin; APP, OVX rats fed a high fat diet with 2 % *Artemisia princeps Pamp* water extract; LJH, OVX rats fed a high fat diet with 2 % *Leonurus japonicas Houtt* water extract; GJE, OVX rats fed a high fat diet with 2 % *Gardenia jasminoides* Ellis water extract; positive-control, OVX rats fed a high fat diet with 30 μg/kg body weight 17β-estradiol + 2 % dextrose. Values are means ± SD

^a,b,c^ Significantly different among all groups by Tukey test at *p* < 0.05

### Energy metabolism

OVX rats had greater weight gain than OVX rats administered with 17β-estrogen (positive-control). Peri-uterine and retroperitoneum fat pads were also higher in OVX rats than in the positive-control rats (Table 2). Despite higher visceral fat, OVX rats had lower serum leptin levels than OVX rats treated with 17β-estradiol (Table 2). Since 17β-estradiol has some adverse effects, alternative therapy needs to alleviate the deterioration of energy metabolism. APP + LJH + GJE, but not APP and APP + LJH treatments, suppressed the increase of body weight in OVX rats as much as the positive-control group (Table 2). Furthermore, peri-uterine and retroperitoneum fat pad weights were lower with APP, APP + LJH and APP + LJH + GJE treatments than the control group, with APP + LJH + GJE having a similar effect as the positive-control (Table 2). Unlike visceral fat amounts, APP + LJH + GJE had serum leptin to levels similar to the positive-control (Table 2). These results indicated that estrogen plays an important role in leptin secretion and that estrogen deficiency induced the impairment of leptin secretion.

Body weight and body fat are balanced by the sum of energy intake and energy expenditure. Food intake was not significantly different among the groups and the rats in all groups had about 20 g/day. Based on the food intake, the daily consumption of herbal extracts was calculated in rats. When the contents were adjusted to human equivalence using the conversion coefficient of 6.2 suggested by the US FDA [23], the daily amount for humans was approximately 3–5 g of the mixture of herbal water extracts. The increase of body weight and visceral fat in OVX rats was primarily due to lower energy expenditure without the modulation of energy intake (Table 3). Furthermore, in OVX rats, carbohydrate oxidation was higher but fat oxidation was lower than the positive-control (Table 3). None of the treatments altered the energy intake in OVX rats.

**Table 3:** Parameters of indirect calorimetry at the end of experiment

	Control (*n* = 12)	APP (*n* = 12)	APP + LJH (*n* = 12)	APP + LJH + GJE (*n* = 12)	Positive-control (*n* = 12)
Caloric intakes (Kcal/day)	95.4 ± 10	104 ± 11	103 ± 11	103 ± 10	92 ± 10
Energy expenditure (kcal/ kg^0.75^/day)	106 ± 12^c^	111 ± 11^bc^	119 ± 12^b^	137 ± 13^a^	131 ± 13^a^
Respiratory quotient	0.84 ± 0.11	0.81 ± 0.10	0.82 ± 0.09	0.80 ± 0.09	0.79 ± 0.09
Carbohydrate oxidation (mg/ kg^0.75^/min)	5.3 ± 0.7^a^	4.3 ± 0.6^b^	4.8 ± 0.6^ab^	4.5 ± 0.5^b^	4.1 ± 0.6^b^
Fat oxidation (mg/ kg^0.75^/min)	6.2 ± 0.8^c^	7.6 ± 0.8^b^	8.0 ± 0.9^b^	10.3 ± 1.2^a^	9.7 ± 1.1^a^

Control, OVX rats fed a high-fat diet (OVX-CON) with 2 % dextrin; APP, OVX rats fed a high fat diet with 2 % *Artemisia princeps Pamp* water extract; LJH, OVX rats fed a high fat diet with 2 % *Leonurus japonicas Houtt* water extract; GJE, OVX rats fed a high fat diet with 2 % *Gardenia jasminoides* Ellis water extract; positive-control, OVX rats fed a high fat diet with 30 μg/kg body weight 17β-estradiol + 2 % dextrose. Values are mean ± SD

^a,b,c^ Significantly different among all groups by Tukey test at *p* < 0.05

APP + LJH and APP + LJH + GJE had greater energy expenditure among OVX rats than the control and the daily energy expenditure of rats in the APP + LJH + GJE group was similar to that of the positive-control group (Table 3). In daily energy expenditure, carbohydrate oxidation was greater in the control rats than in the positive-control rats whereas fat oxidation contrasted with carbohydrate oxidation (Table 3). APP alone caused greater carbohydrate and fat oxidation in OVX rats whereas the APP + LJH + GJE group had the greatest carbohydrate and fat oxidation and reached the same amount as the positive-control (Table 3).

### Body composition

**Fig. 2:**
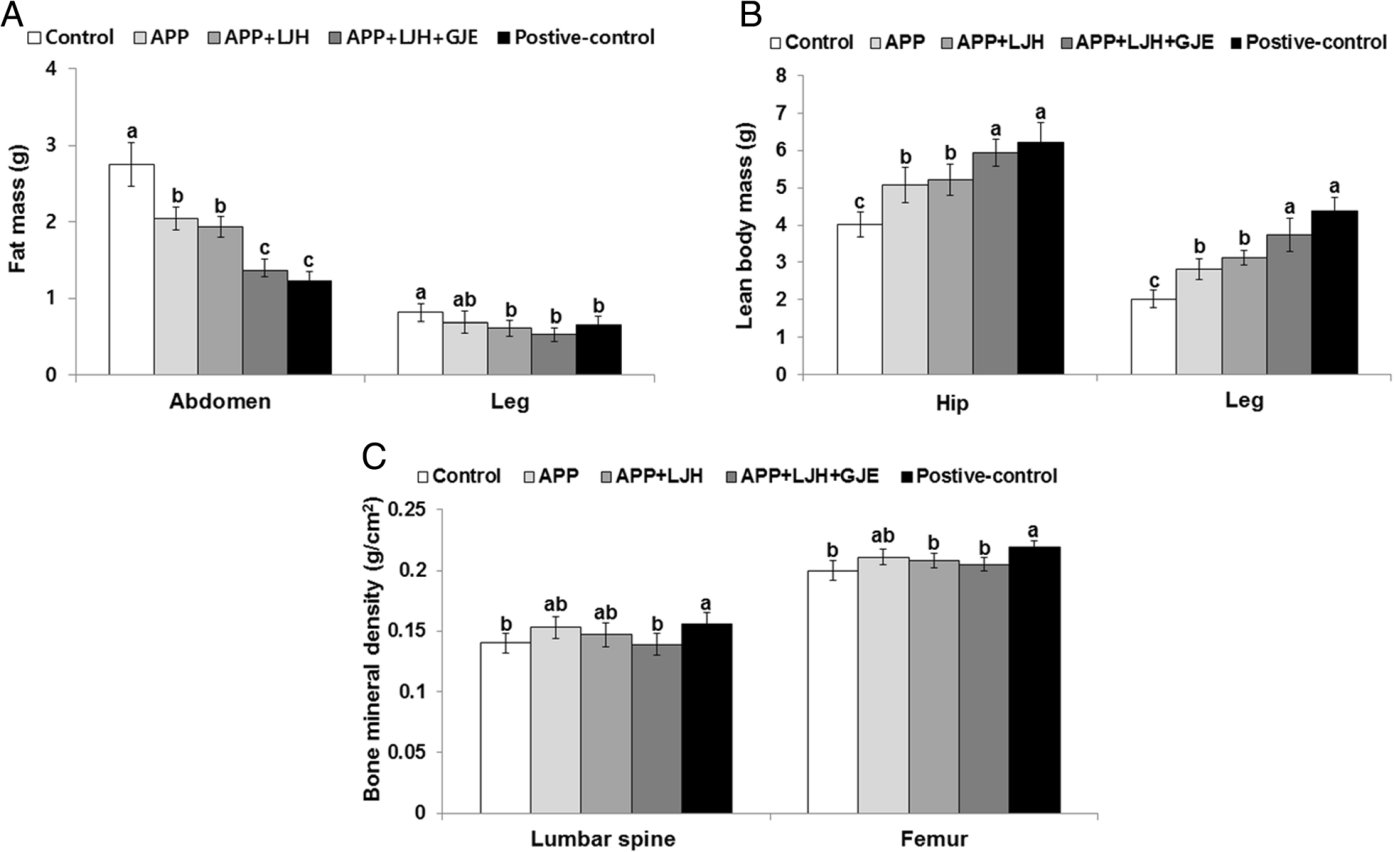
Body composition at 8th weeks of experimental period measured by DEXA. Control, OVX rats fed a high-fat diet with 2 % dextrin; APP, OVX rats fed a high fat diet with 2 % *Artemisia princeps Pamp* water extract; LJH, OVX rats fed a high fat diet with 2 % *Leonurus japonicas Houtt* water extract; GJE, OVX rats fed a high fat diet with 2 % *Gardenia jasminoides* Ellis water extract; positive-control, OVX rats fed a high fat diet with 30 μg/kg body weight 17β-estradiol + 2 % dextrose. At 8th weeks, lean body mass (**a**) and fat mass (**b**) were measured in the abdomen and leg by DEXA whereas bone mineral density (BMD) of the femur and lumbar spine (**c**) were also measured. Bars and error bars represent means ± SD (*n* = 12). ^a,b,c^ Significantly different among all groups by Tukey’s test at *p* < 0.05

Consistent with the amounts of peri-uterine and retroperitoneum fat pads, the fat mass in the abdomen and leg measured by DEXA was significantly higher in the control rats than in the positive-control rats (Fig 2a). The fat mass in the abdomen was lowered in the descending order of the control < APP = APP + LJH < APP + LJH + GJE = positive-control. APP + LJH + GJE treatment was the only herbal treatment that reduced fat mass in the leg and the reduction was lower than in the positive-control (Fig. 2a).

In contrast to the fat mass, lean body mass in the abdomen and leg was lower in the control rats than in the positive-control rats. The lower lean body mass was reduced by APP, APP + LJH, and APP + LJH + GJE in OVX rats and the lean body mass in the abdomens and legs of the APP + LJH + GJE group was similar to that of the positive-control group (Fig. 2b). APP suppressed the decrease of lean body mass in the leg as much as the APP + LJH + GJE and positive-control groups (Fig. 2b).

BMD in the lumbar spine and leg was lower in the control rats than in the positive-control rats (Fig. 2c). The decrease of BMD in OVX rats was attenuated by APP but the decrease was not significant. Other herbal extracts did not alter the BMD in OVX rats (Fig. 2c).

### Lipid profiles and liver function index

Serum levels of total and LDL cholesterol and triglyceride were higher in control rats than in 17β-estradiol-treated rats whereas HDL cholesterol levels in the circulatory system were lower in control rats (Table 4). APP and APP + LJH treatments suppressed the elevation of serum LDL cholesterol levels and only APP + LJH + GJE and normalized the lower of serum HDL cholesterol to levels similar to the positive-control group. APP, APP + LJH and APP + LJH + GJE all resulted in lower serum triglyceride levels than the control group (Table 4). Thus, all the treatments exhibited some beneficial effects on lipid metabolism in the circulatory system of OVX rats.

**Table 4:** Lipid profiles, liver function index and serum glucose and insulin levels in overnight-fasted rats

	Control (*n* = 12)	APP (*n* = 12)	APP + LJH (*n* = 12)	APP + LJH + GJE(*n* = 12)	Positive-control (*n* = 12)
Total cholesterol (mg/dL)	109.9 ± 10.3^a^	101.3 ± 10.4^ab^	104.5 ± 10.4^ab^	108.5 ± 10.9^a^	99.4 ± 9.5^b^
LDL cholesterol (mg/dL)	56.3 ± 6.4^a^	50.7 ± 5.7^b^	51.6 ± 5.8^b^	53.4 ± 7.4^ab^	45.2 ± 5.6^b^
HDL cholesterol (mg/dL)	32.3 ± 3.3^b^	34.2 ± 3.7^ab^	35.7 ± 3.8^ab^	36.9 ± 3.6^a^	38.3 ± 3.7^a^
Triglyceride (mg/dL)	107.1 ± 10.4^a^	81.4 ± 8.8^b^	86.1 ± 9.8^b^	80.3 ± 8.1^b^	79.6 ± 8.5^b^
Aspartate aminotransferase (IU/L)	123 ± 9^a^	111 ± 12^ab^	109 ± 14^b^	90 ± 15^c^	87 ± 14^c^
Alanine aminotransferase (IU/L)	39.8 ± 4.6	41.5 ± 5.9	40.1 ± 6.8	35.6 ± 5.2	35.5 ± 5.5
Glucose (mg/dL)	100.3 ± 11.4^a^	87.5 ± 10.4^b^	92.8 ± 11.3^ab^	93.0 ± 10.8^ab^	94.9 ± 10.7^ab^
Insulin (ng/mL)	1.87 ± 0.28^a^	1.29 ± 0.25^b^	1.30 ± 0.24^b^	1.15 ± 0.22^c^	1.14 ± 0.25^c^
HOMA-IR	10.4 ± 1.8^a^	6.3 ± 0.8^bc^	6.7 ± 0.8^b^	5.9 ± 0.7^c^	6.0 ± 0.7^c^

Control, OVX rats fed a high-fat diet (OVX-CON) with 2 % dextrin; APP, OVX rats fed a high fat diet with 2 % *Artemisia princeps Pamp* water extract; LJH, OVX rats fed a high fat diet with 2 % *Leonurus japonicas Houtt* water extract; GJE, OVX rats fed a high fat diet with 2 % *Gardenia jasminoides* Ellis water extract; positive-control, OVX rats fed a high fat diet with 30 μg/kg body weight 17β-estradiol + 2 % dextrose. HOMA-IR, homeostasis model assessment estimate of insulin resistance. Values are means ± SD

^a,b,c^ Significantly different among all groups by Tukey test at *p* < 0.05

The major adverse effects of herbal treatments are generally liver damage. AST and ALT are found in various body tissues, and the elevation of serum ALT and AST levels are clinically used as a part of a diagnostic evaluation of hepatocellular injury. Serum AST levels were higher in the control rats than the positive-control rats and they decreased in the descending order of control, APP, APP + LJH, APP + LJH + GJE, and positive-control. APP + LJH + GJE lowered them as much as the positive-control group (Table 4). However, serum ALT levels were not significantly different between the control and positive-control groups and APP, APP + LJH, and APP + LJH + GJE did not modify serum ALT levels (Table 4).

### Glucose metabolism

Overnight-fasted serum glucose levels were higher in OVX rats in comparison to positive-control rats but not significantly (Table 4). However, the levels were significantly lower in APP rats than the control rats. Serum insulin levels in overnight-fasting states were much higher in OVX rats than in positive-control rats, but were lower in all OVX rats given herbal extract treatments than the control rats (Table 4). HOMA-IR, an index of insulin resistance, was higher in the OVX group than in the positive-control group. HOMA-IR was lower in the descending order of the control > APP + LJH > APP > APP + LJH + GJE > positive-control groups (Table 4). APP + LJH + GJE lowered the elevation of HOMA-IR similar to the positive-control group.

**Fig. 3:**
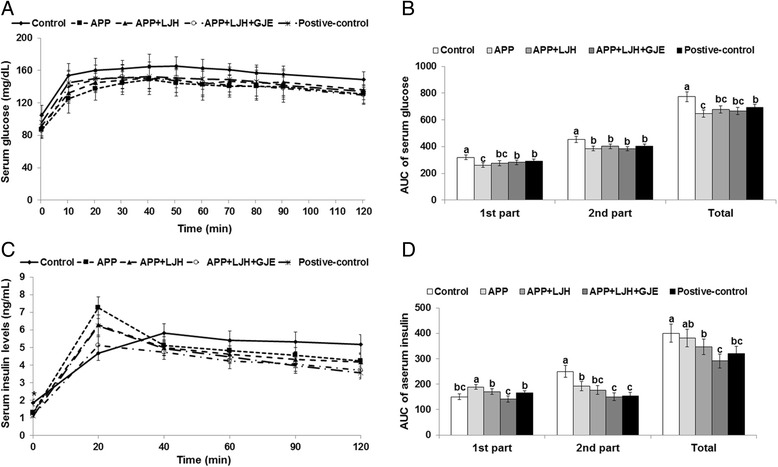
Serum glucose levels and area under the curve of glucose and insulin during oral glucose tolerance test (OGTT). Control, OVX rats fed a high-fat diet with 2 % dextrin; APP, OVX rats fed a high fat diet with 2 % *Artemisia princeps Pamp* water extract; LJH, OVX rats fed a high fat diet with 2 % *Leonurus japonicas Houtt* water extract; GJE, OVX rats fed a high fat diet with 2 % *Gardenia jasminoides* Ellis water extract; positive-control, OVX rats fed a high fat diet with 30 μg/kg body weight 17β-estradiol + 2 % dextrose. At 7th week, an OGTT was conducted in overnight-fasted animals by orally administering 2 g glucose/kg body weight. After glucose loading, blood samples were taken by tail bleeding at 0, 10, 20, 30, 40, 50, 60, 70, 80, 90, and 120 min to measure serum glucose levels (**a**) and serum insulin levels were measured at 0, 20, 40, 60, 90 and 120 min (**c**). Serum glucose levels had significant time and treatment effects but not interaction effects (*P* < 0.05) in two-way repeated measures ANOVA whereas serum insulin levels exhibited significant effect of time and treatment effects and their interaction effect (*P* < 0.05). During OGTT area under the curve (AUC) of serum glucose (**b**) and insulin (**d**) in the first part (0–40 min), second part (40–120 min) and total parts were given. Bars or dots and error bars represent means ± SD (*n* = 12). ^a,b,c^ Significantly different among all groups by Tukey’s test at *p* < 0.05

OGTT indicates the relative roles of insulin secretion and insulin resistance in the progression of glucose intolerance. Serum glucose levels were overall higher in OVX rats than in positive-control rats at all OGTT time points (Fig. 3a). Two-way repeated measures ANOVA revealed that serum glucose levels during OGTT has significant effects of time and treatment (*P* < 0.05) but there were no interaction effects. APP + LJH and APP + LJH + GJE did not increase serum glucose levels at 10–40 min as much as the levels of the OVX-control group (Fig. 3a). Serum glucose levels at 10–40 min were higher in the ascending order of APP < APP + LJH < APP + LJH + GJE = positive control < control in OVX rats during OGTT (Fig. 3a). After peaking, serum glucose levels gradually decreased, with APP + LJH + GJE and the positive control exhibiting a faster decrease than the control (Fig. 3a). Serum glucose levels increased up to 40–50 min after glucose challenge whereas after that point serum glucose levels gradually decreased. Thus, area under the curves of glucose (AUCG) and insulin (AUCI) were separately calculated into the two parts in the 1st part (0–40 min) and 2nd parts (40-120 min). The AUCGs of the 1st and 2nd parts were much greater in the control group than in the positive-control group (Fig. 3b). The AUCGs of the 1st part were lowest in the APP group and was similar in both the APP + LJH + GJE and positive-control groups (Fig. 3b). Serum insulin levels during OGTT are shown in Fig. 3c. Serum insulin levels increased until 20 min and then they were lowered in herbal treatment groups and positive-control group but they were increased until 40 min and then slowly decreased in the control group (Fig. 3c). Two-way repeated measures ANOVA demonstrated that there was a significant effect of time and treatment effects and their interaction effect (*P* < 0.05). Serum insulin levels increased until 40 mins and slightly decreased after 40 mins in OVX-control rats but in herbal treatment groups serum insulin levels peaked at 20 min and then they markedly decreased. AUCIs of the 1st part were higher in the ascending order of APP + LJH + GJE < control < APP + LJH = positive-control < APP (Fig. 3d). The AUCGIs of the 2nd part were highest in the control and APP groups, lower in the APP + LJH group and even lower in the APP + LJH + GJE and positive-control groups (Fig. 3d). Thus, the serum glucose levels in the 1st part were mainly associated with serum insulin levels.

**Fig. 4:**
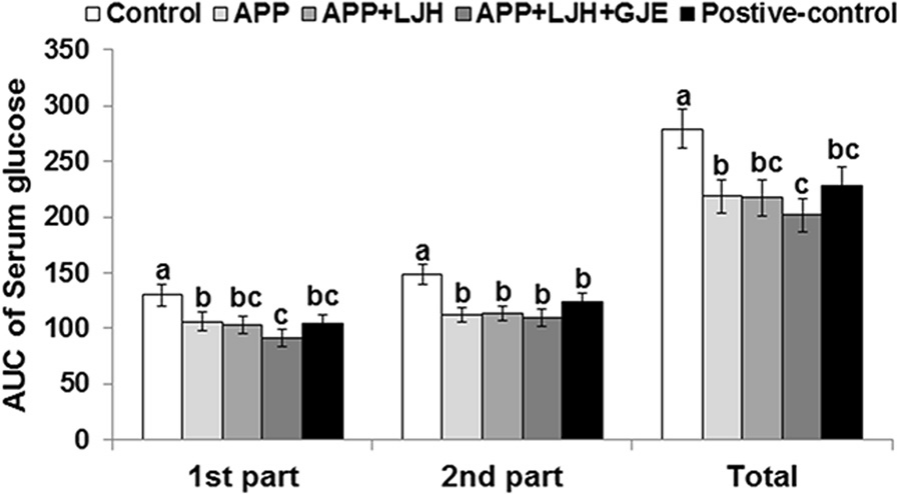
Serum glucose levels and area under the curve of glucose and insulin during insulin tolerance test (ITT). Control, OVX rats fed a high-fat diet with 2 % dextrin; APP, OVX rats fed a high fat diet with 2 % *Artemisia princeps Pamp* water extract; LJH, OVX rats fed a high fat diet with 2 % *Leonurus japonicas Houtt* water extract; GJE, OVX rats fed a high fat diet with 2 % *Gardenia jasminoides* Ellis water extract; positive-control, OVX rats fed a high fat diet with 30 μg/kg body weight 17β-estradiol + 2 % dextrose. At three days after OGTT, after the withdrawal of food for 6 h serum glucose levels were measured every 15 min for 90 min after intraperitoneal insulin injection (0.75 U/kg body weight). During ITT area under the curve (AUC) of serum glucose in the first part (0–30 min) and second part (30–90 min). Bars and error bars represent means ± SD (*n* = 12). ^*^Significantly different among groups in one-way ANOVA at *p* < 0.05. ^a,b,c^ Significantly different among all groups by Tukey’s test at *p* < 0.05

Insulin resistance can be determined by ITT. In ITT, the decrease of serum glucose levels during the 1st part (0–30 min) of AUC was related to insulin resistance. In the 2nd part (40–90 min) of ITT, serum glucose levels were maintained and slowly increased. The AUC of serum glucose levels in the 1st and 2nd parts of the ITT were higher in the control group than the positive-control group and APP + LJH + GJE decreased the 1st part the most (Fig. 4). The 2nd part of AUC was higher in the control group than the positive-control group. APP, APP + LJH and APP + LJH + GJE had similar AUCs in the 2nd part as the positive-control during ITT (Fig. 4). These results indicated that APP + LJH + GJE reduced insulin resistance the most.

### Lipid in the liver and skeletal muscles

Since a high fat diet induces insulin resistance by increasing intracellular triglyceride storage, intracellular triglyceride contents were measured in the liver and skeletal muscle. The contents of hepatic triglyceride storage were higher in the control group than in the positive-control group (Table 5). The storage was lower in the descending order of the control < APP = APP + LJH < APP + LJH + GJE = positive-control. The gastrocnemius and quadriceps muscles, the major skeletal muscles in the leg, stored more triglyceride in the control rats than in the positive-control rats. The lower triglyceride storage in skeletal muscles of rats given APP + LJH + GJE were similar to that of the positive-control group (Table 5).

**Table 5:** Triglyceride storage in the liver, gastrocnemius muscle and quadriceps muscles

	Control (*n* = 12)	APP (*n* = 12)	APP + LJH (*n* = 12)	APP + LJH + GJE(*n* = 12)	Positive-control (*n* = 12)
Liver (mg/g tissues)	0.86 ± 0.10^a^	0.75 ± 0.09^b^	0.74 ± 0.07^b^	0.61 ± 0.09^c^	0.57 ± 0.08^c^
Gastrocnemius muscles (mg/g tissues)	1.25 ± 0.21^a^	1.18 ± 0.18^ab^	1.10 ± 0.22^ab^	1.02 ± 0.17^b^	0.93 ± 0.17^b^
Quadriceps muscles (mg/g tissues)	2.62 ± 0.39^a^	2.24 ± 0.36^b^	2.18 ± 0.32^bc^	2.01 ± 0.28^c^	1.93 ± 0.29^c^

Control, OVX rats fed a high-fat diet (OVX-CON) with 2 % dextrin; APP, OVX rats fed a high fat diet with 2 % *Artemisia princeps Pamp* water extract; LJH, OVX rats fed a high fat diet with 2 % *Leonurus japonicas Houtt* water extract; GJE, OVX rats fed a high fat diet with 2 % *Gardenia jasminoides* Ellis water extract; positive-control, OVX rats fed a high fat diet with 30 μg/kg body weight 17β-estradiol + 2 % dextrose. Values are means ± SD

^a,b,c^ Significantly different among all groups by Tukey test at *p* < 0.05

### Expression of genes related fatty acid oxidation and synthesis and hepatic insulin signaling

**Fig. 5:**
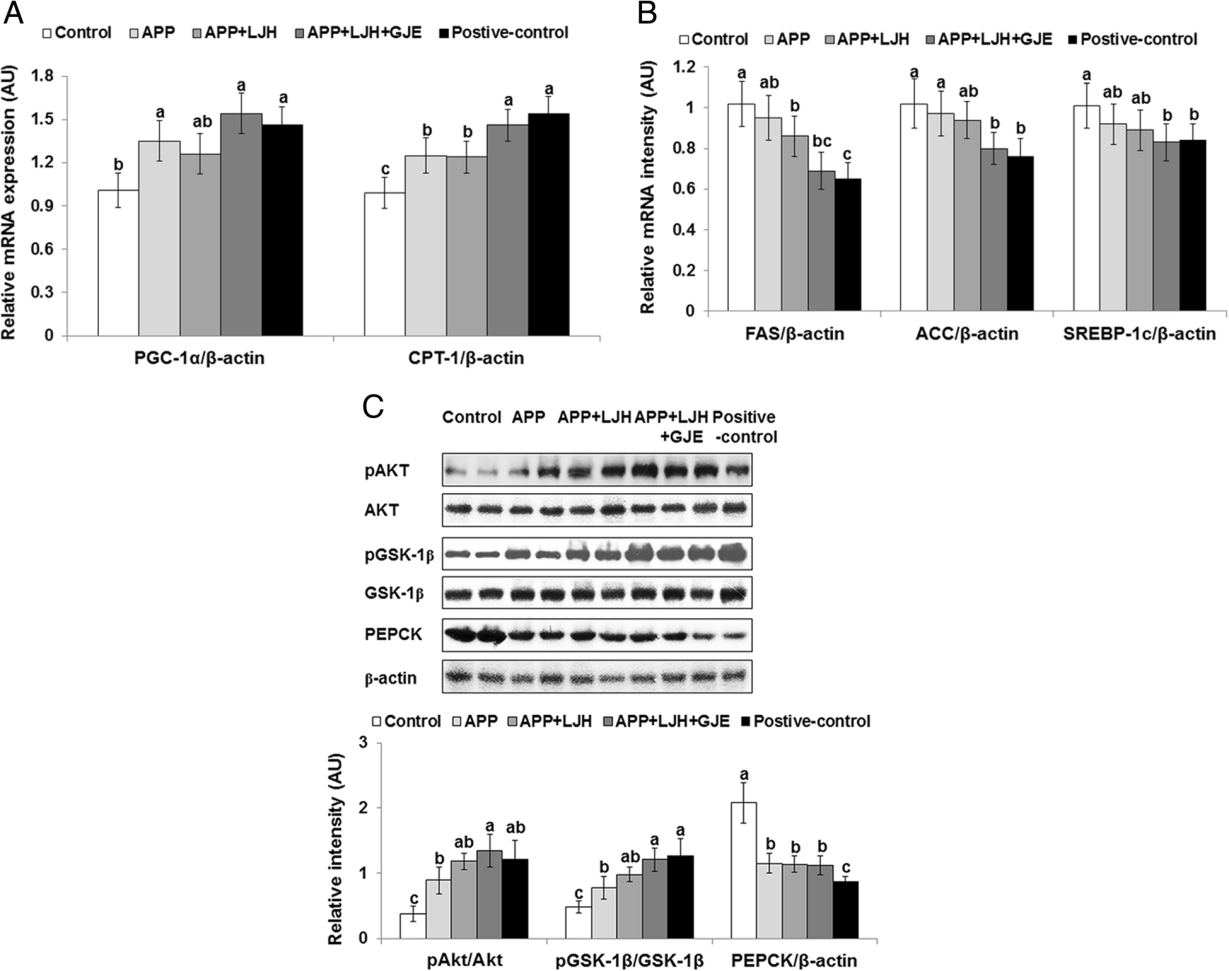
The mRNA expression of genes related to fatty acid metabolism and insulin signaling in the liver. Control, OVX rats fed a high-fat diet with 2 % dextrin; APP, OVX rats fed a high fat diet with 2 % *Artemisia princeps Pamp* water extract; LJH, OVX rats fed a high fat diet with 2 % *Leonurus japonicas Houtt* water extract; GJE, OVX rats fed a high fat diet with 2 % *Gardenia jasminoides* Ellis water extract; positive-control, OVX rats fed a high fat diet with 30 μg/kg body weight 17β-estradiol + 2 % dextrose. At end of the experimental period, mRNA levels of hepatic genes involved in fatty acid oxidation (**a**) and synthesis (**b**) were measured by real-time PCR. Insulin signaling (**c**) also measured by immunoblotting assay. Bars and error bars represent means ± SD (*n* = 6). ^a,b,c^ Significantly different among all groups by Tukey’s test at *p* < 0.05

Since triglyceride storage is the net of fatty acid oxidation and synthesis, the expression of genes involved in oxidation and synthesis were measured. The metabolism of fatty acids was associated with PGC-1α in the liver. The expression of PGC-1α was lower in the control rats than other treatment groups and APP + LJH + GJE increased PGC-1α expression in comparison to the control group (Fig. 5a). In parallel with the modulation of PGC-1α expression, hepatic expression of CPT-1, the mitochondrial transporter of fatty acids and a major regulator of fatty acid oxidation, was lower in control rats than in positive-control rats (Fig. 5a). This result indicated that fatty acid oxidation increased in APP + LJH + GJE compared to the control group. The expressions of hepatic SREBP-1c, FAS and ACC, which are regulatory enzymes of fatty acid synthesis, were higher in control rats than the positive-control rats (Fig. 5b). In OVX rats given APP + LJH + GJE CPT-1 expression was higher and FAS expression was lower; SREBP-1c and ACC to levels similar to those of the positive-control rats (Fig. 5b).

In hepatic insulin signaling, the phosphorylation of Akt was lower in the control group than in the positive-control and APP groups. APP + LJH and APP + LJH + GJE treatments increased phosphorylated Akt in OVX rats (Fig. 5c). In parallel with Akt phosphorylation, the phosphorylation of GSK-1β was lower in control rats than positive-control rats whereas APP + LJH + GJE elevated the phosphorylated GSK-1β (Fig. 5c).

## Discussion

Estrogen deficiency deteriorates energy, lipid, glucose and bone metabolism. Hormonal therapy is known to be lacking in terms of preventing the deterioration and has been reported to cause adverse effects [1, 4, 24]. Alternative treatments have been researched and some herbal extracts have been successfully employed [25]. In Korea, herbs such as APP and LJH were traditionally used for enhancing women’s health [8, 10] and they can be beneficial for menopausal symptoms. APP, LJH, and GJE are traditionally used to improve women’s health. The present study found that the herbal extracts attenuated various menopausal symptoms and their possible action mechanisms were examined. APP + LJH + GJE appeared to improved energy balance by increasing energy expenditure as much as estrogen treatment. Furthermore, APP + LJH + GJE treated OVX rats had less visceral and intramuscular fat and greater lean body mass and were similar to the positive-control. APP itself was sufficient to improve lipid profiles in the circulatory system and to elevate glucose-stimulated insulin secretion. APP + LJH + GJE also attenuated dyslipidemia while improving glucose intolerance and insulin resistance in OVX rats. The expressions of hepatic genes involved in fatty acid synthesis were lower in the APP + LJH + GJE group in comparison to the control group, which may improve lipid and glucose metabolism. These results suggested that the combination of APP + LJH + GJE may have potential as a therapeutic agent for the treatment of postmenopausal symptoms.

Estrogen plays an important role in maintaining energy, glucose, lipid and bone metabolism [2, 3]. As expected, estrogen deficiency offsets the estrogen effects in these processes, which leads to metabolic disorders such as insulin resistance, obesity, dyslipidemia, high blood pressure and hyperglycemia. Epidemiologic studies have demonstrated that overweight and obesity rates are increased in menopausal women and that obesity is closely related to the risk of metabolic syndromes [26, 27]. However, the cause of obesity in menopausal women remains unclear. Estrogen deficiency plays an important role in developing menopausal symptoms including gaining weight but it cannot explain them completely. Furthermore, estrogen treatment only partly reduces metabolic disturbances in menopausal women but exacerbates some cardiovascular diseases such as stroke [28]. Therefore, the discrepancy in the metabolic effects of hormone therapy needs to be explained. The present study suggested that estrogen deficient rats experienced the reduction of energy expenditure without changing food intake and the increase of visceral fats by decreasing fatty acid oxidation. In parallel with increasing visceral fat mass, dyslipidemia and hyperglycemia were exhibited in OVX rats in comparison to the positive-control. The previous studies have exhibited that sham-operated rats have a similar metabolism of energy, glucose, lipid and bone as the positive-control rats [7, 29]. Thus, the results in the present study indicated that OVX rats had impaired energy, glucose, lipid and bone metabolism and estrogen treatment attenuated the deterioration of the metabolism in OVX rats. As life expectancy increases, women are substantially influenced by menopausal symptoms for a significant period. As a result, estrogen deficiency may increase the chance to develop metabolic diseases in post-menopausal women.

Estrogen increases PGC-1α expression via nitric oxide/cGMP signaling pathways through estrogen receptors which enhances lipid, energy and glucose metabolism [28, 30]. Overnutrition suppresses the expression of PGC-1α [31]. Thus, both overnutrition and estrogen deficiency suppress the transcription of genes related to metabolic and mitochondrial function such as nuclear respiratory factors, peroxisomal proliferator-activated receptors (PPARs) and cAMP responding element binding protein [28, 30, 31]. The inhibition of these signals decreases oxidative phosphorylation, lipid oxidation and insulin sensitivity [31]. Thus, the activation of PGC-1α signaling attenuates menopausal symptoms without direct activation of the estrogen receptor by estrogen. The present study showed similar results in OVX rats, as the expression of PGC-1α was lower, whereas it was higher with APP and APP + LJH + GJE treatments. The lower PGC-1α expression in OVX rats increased the expression of genes involved in fatty acid synthesis. Thus, APP and APP + LJH + GJE potentiated PGC-1α expression and improved dyslipidemia and hyperglycemia. These results indicated that the improvement of PGC-1α may attenuate menopausal symptoms.

APP, LJH and GJE have not been evaluated for their effects on anti-menopausal symptoms in experimental animal and human studies. APP and its essential oils have been reported to have anti-oxidant, anti-microbial (eucalyptol and α-terpineol) and anti-thrombotic (sulfated polysaccharides) activities [8, 9]. Some of these activities are associated with the inhibition of the expression of proinflammatory cytokines through the activation of NF-κB pathways [9]. In the present study, APP ameliorated the suppressed glucose metabolism due to estrogen deficiency in rats. APP also potentiated glucose-stimulated insulin secretion and attenuated insulin resistance measured by HOMA-IR. However, it did not inhibit the deterioration of energy and lipid metabolism in OVX rats as much as the positive-control. Thus, the combined mixture is beneficial for reducing various menopausal symptoms without increasing uterine proliferation.

In the estrogen deficient state, glucose metabolism is deteriorated by increased insulin resistance and menopausal women with low insulin secretion capacity are susceptible to type 2 diabetes [2, 3]. Insulin resistance develops mainly in the liver, skeletal muscle, and adipose tissue. In the insulin resistant state, myocytes in skeletal muscle exhibit reduced glycogen synthesis [32] and glycogenolysis and gluconeogenesis in the liver are increased even in hyperinsulinemic states [33]. Previous studies have demonstrated that OVX rats impaired glucose metabolism by increasing insulin resistance and deteriorating the regulation of insulin secretion in comparison to the sham-operated rats [14, 29]. Thus, estrogen deficiency develops insulin resistance even though overnight fasted serum glucose levels are normal. The present study indicated that OVX rats had insulin resistance. APP + LJH + GJE markedly lowered serum glucose levels more than the other treatments in the early part of ITT although rats with all treatments had lower HOMA-IR, an insulin resistance index. The results also indicated that APP + LJH + GJE treated and positive control rats had the best insulin sensitivity in the hyperinsulinemic state. This was consistent with the potentiation of Akt and GSK-1β phosphorylation and with the lower PEPCK expression. Therefore, APP + LJH + GJE can be a good candidate for improving insulin sensitivity in post-menopausal women.

LJH and GJE extracts were selected as additional herbs with APP for the purpose of attenuating menopausal symptoms. LJH has not been studied previously, although it is a folk medicine for reducing primary dysmenorrhea by enhancing blood circulation in Korea. In Chinese medicine, APP is often used with LJH to synergistically improve the function of both herbs [10]. Since APP itself did not reduce energy and lipid metabolism in OVX rats, GJE was added to the mixture in the present study. GJE and its major components such as geniposides, genipin and crocetin have been studied previously with respect to various functions [12, 34–36]. They exhibited anti-inflammatory activities to ameliorate atopic dermatitis and arthritis and have anti-depressant, anti-oxidant, anti-hypertensive and hypoglycemic activities [35, 36]. Chen et al. [12] demonstrated that GJE activates PPAR-γ to induce adipocyte differentiation, decreases glucose release from the liver and enhances glycogen storage in skeletal muscle cells. This hypoglycemic effect is reported to be similar to thiazolidinediones, known as PPAR-γ activators, by stimulating of PPAR-γ activity and/or its expression to increase glucose uptake in adipose tissues [12]. In the present study, APP improved glucose tolerance by increasing insulin secretion in the first part of OGTT, but also lowered insulin sensitivity more than the control. However, APP + LJH + GJE increased insulin sensitivity more than APP alone during ITT. This was associated with the decrease of hepatic PEPCK expression, a regulatory enzyme for gluconeogenesis, potentiating the phosphorylation of GSK-1β, which is involved in insulin signaling. In addition, treating with both APP and APP + LJH + GJE resulted greater liver expression of PGC-1α, the upstream modulator of PPAR-γ. Not only GJE but also APP may improve glucose metabolism by potentiating hepatic insulin signaling. Therefore, APP + LJH + GJE may alleviate the impairment of hepatic glucose metabolism due to estrogen deficiency in menopausal women.

Potential toxicity of dietary and medicinal herbs need to be evaluated prior to their use. Although some plants of the genus *Artemesia* are known to be toxic, *Artemisia princeps* is a commonly used herb in traditional medicines, teas, and as a cooked green vegetable. It is rich in a variety of nutrients and is generally considered to a safe food [37]. *Leonurus japonicas* is a very commonly used herb in Oriental Medicine with a well-established record for safe use; one of its valuable properties is as a hepatoprotective intervention against liver toxins [38]. The ripe fruits of *Gardenia jasminoides* contain geniposide, an iridoid glycoside, which was determined in this study. A toxicology study established the LD50 of geniposide to be 1431.1 mg/kg body weight and liver toxicity was seen at 574 mg/kg [39]. When geniposide was administered at 24.3 and 72.9 mg/kg body weight for 90 days there was no indication of toxicity. The doses used in this study would only result in doses of about 1 mg/kg body weight. Therefore, it is apparent that the doses used in this study were within a very safe range. Nevertheless, it is important to be aware of its potential toxicity at higher doses, and possibly in individuals with greater sensitivity. In the present study no rats exhibited any side-effect of APP, LJH and GJE at the doses they were given. No rats died during the experimental period and no organs had apparent damage when they were examined. Serum ALT and AST levels in the treatment groups were lower than the control group, indicating no hepatotoxicity. Furthermore, these herbs have not been reported to have side effects and toxicity and they are registered as foods by the Korean Food and Drug Administration. However, since they have been demonstrated to cause apoptosis of carcinoma cells [37, 38], they might have some toxicity.

## Conclusions

APP alone improved dyslipidemia and glucose intolerance by potentiating glucose-stimulated insulin secretion as much as APP + LJH + GJE in OVX rats. However, energy expenditure, fatty acid oxidation and insulin resistance was inhibited by APP + LJH + GJE as much as the positive-control. APP and APP + LJH + GJE potentiated PGC-1α expression in the liver and improved glucose tolerance in estrogen deficient rats. APP + LJH + GJE inhibited gluconeogenesis in the fed state by suppressing PEPCK expression. These results suggested that the combination of APP + LJH + GJE attenuated various menopausal symptoms in OVX rats. Thus, it may have potential as a therapeutic agent for the treatment of postmenopausal symptoms.

### Open access

This article is distributed under the terms of the Creative Commons Attribution 4.0 International License (http://creativecommons.org/licenses/by/4.0/), which permits unrestricted use, distribution, and reproduction in any medium, provided you give appropriate credit to the original author(s) and the source, provide a link to the Creative Commons license, and indicate if changes were made. The Creative Commons Public Domain Dedication waiver (http://creativecommons.org/publicdomain/zero/1.0/) applies to the data made available in this article, unless otherwise stated.

## Abbreviation

APP: *Artemisia princeps Pamp*
LJH: *Leonurus japonicas Houtt*
GJE: *Gardenia jasminoides Ellis*
OVX: ovariectomy
OGTT: oral glucose tolerance
VO_2_: average oxygen consumption
VCO_2_: average carbon dioxide production
PGC-1α: peroxisome proliferator-activated receptor-γ coactivator-1α
CPT-1: carnitine palmitoyltransferase-1
ACC: acetyl CoA carboxylase
SREBP-1c: sterol regulatory element-binding protein-1c
FAS: fatty acid synthase
En%: energy percent
ITT: insulin tolerance test
DEXA: dual-energy X-ray absorptiometry
BMD: bone mineral density
AST: aspartate aminotransferase
ALT: alanine aminotransferase
HOMA-IR: homeostasis model assessment estimate of insulin resistance
AUCG: area under the curves of glucose
AUCI: area under the curves of insulin
ANOVA: analysis of variance
PPAR: peroxisomal proliferator-activated receptor
